# School-based surveys of malaria in Oromia Regional State, Ethiopia: a rapid survey method for malaria in low transmission settings

**DOI:** 10.1186/1475-2875-10-25

**Published:** 2011-02-03

**Authors:** Ruth A Ashton, Takele Kefyalew, Gezahegn Tesfaye, Rachel L Pullan, Damtew Yadeta, Richard Reithinger, Jan H Kolaczinski, Simon Brooker

**Affiliations:** 1 Malaria Consortium Ethiopia, PO Box 110224, Ethio-China Road, Addis Ababa, Ethiopia; 2 Malaria Consortium Africa, PO Box 8045, Plot 25, Upper Naguru East Road, Kampala, Uganda; 3 London School of Hygiene and Tropical Medicine, Keppel Street, London, WC1E 7HT, UK; 4 Oromia Regional Health Bureau, PO Box 24341, Addis Ababa, Ethiopia; 5 U.S. Agency for International Development, Addis Ababa, Ethiopia; 6 Kenya Medical Research Institute-Wellcome Trust Research Programme, PO Box 43640-00100, Nairobi, Kenya

## Abstract

**Background:**

In Ethiopia, malaria transmission is seasonal and unstable, with both *Plasmodium falciparum *and *Plasmodium vivax *endemic. Such spatial and temporal clustering of malaria only serves to underscore the importance of regularly collecting up-to-date malaria surveillance data to inform decision-making in malaria control. Cross-sectional school-based malaria surveys were conducted across Oromia Regional State to generate up-to-date data for planning malaria control interventions, as well as monitoring and evaluation of operational programme implementation.

**Methods:**

Two hundred primary schools were randomly selected using a stratified and weighted sampling frame; 100 children aged five to 18 years were then randomly chosen within each school. Surveys were carried out in May 2009 and from October to December 2009, to coincide with the peak of malaria transmission in different parts of Oromia. Each child was tested for malaria by expert microscopy, their haemoglobin measured and a simple questionnaire completed. Satellite-derived environmental data were used to assess ecological correlates of *Plasmodium *infection; Bayesian geostatistical methods and Kulldorff's spatial scan statistic were employed to investigate spatial heterogeneity.

**Results:**

A total 20,899 children from 197 schools provided blood samples, two selected schools were inaccessible and one school refused to participate. The overall prevalence of *Plasmodium *infection was found to be 0.56% (95% CI: 0.46-0.67%), with 53% of infections due to *P. falciparum *and 47% due to *P. vivax*. Of children surveyed, 17.6% (95% CI: 17.0-18.1%) were anaemic, while 46% reported sleeping under a mosquito net the previous night. Malaria was found at 30 (15%) schools to a maximum elevation of 2,187 metres, with school-level *Plasmodium *prevalence ranging between 0% and 14.5%. Although environmental variables were only weakly associated with *P. falciparum *and *P. vivax *infection, clusters of infection were identified within Oromia.

**Conclusion:**

These findings demonstrate the marked spatial heterogeneity of malaria in Oromia and, in general, Ethiopia, and provide a strong epidemiological basis for planning as well as monitoring and evaluating malaria control in a setting with seasonal and unstable malaria transmission.

## Background

Following the recent achievements in global malaria control [1], there is increased emphasis on monitoring these achievements and on refining the epidemiological landscape in order to determine intervention needs and guide implementation [2]. Household surveys, including Malaria Indicator Surveys (MISs) [3], Demographic Health Surveys [4] and Multiple Indicator Cluster Surveys [5] are commonly used to achieve these surveillance and monitoring goals, but they are expensive, time-consuming and technically complicated to undertake. A complementary, inexpensive framework for malaria surveillance may be provided by school malaria surveys [6], which were an important component of early, particularly colonial, malaria reconnaissance, and more recently have contributed towards a nationwide assessment of malaria in Kenya [7].

Building on the Kenyan experience, this paper presents results from the first, large-scale school survey of malaria in Ethiopia. Malaria transmission in Ethiopia is temporally and spatially dynamic [8], with transmission unstable, seasonal, and linked to environmental variables such as altitude and rainfall [9]. In recent years, there has been a marked scale-up of the distribution of long-lasting insecticidal nets (LLINs) and indoor residual spraying (IRS) in Ethiopia [10]. To track this progress and to capture the inherent heterogeneities of malaria transmission in the country, various community-based malaria surveys have been carried out at regional state and national levels [11-13]. The aim of the present work was to generate data for Oromia Regional State to assist in targeting malaria control interventions across this heterogeneous transmission setting.

## Methods

### Study setting

**Figure 1:**
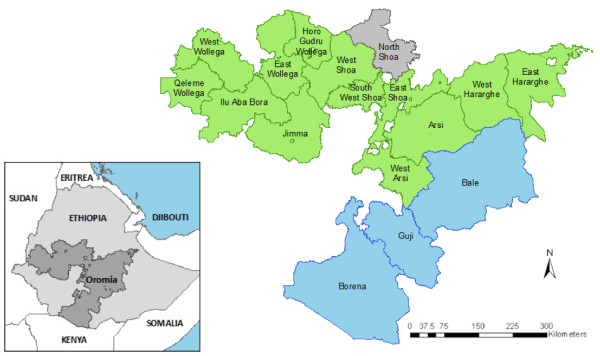
**Location of Oromia Regional State within Ethiopia (inset) and administrative zones surveyed in May 2009 (blue) and October to December 2009 (green)**. North Shoa (grey) was not surveyed as no schools in this zone were randomly selected for inclusion.

This study was undertaken throughout Oromia Regional State, the largest of Ethiopia's 11 regional states. Oromia covers approximately one third of the country's landmass (Figure 1) and has a population of 27 million [14], an estimated 17 million of whom are at risk of malaria [15]. It is divided into 17 administrative zones, as defined in Central Statistics Agency (CSA) 2007 census data [14], with each zone divided further into woredas (i.e. districts) followed by kebeles (i.e. municipalities).

Oromia is geographically diverse, encompassing arid lowlands, fertile and well-vegetated areas with high rainfall, and cool mountainous regions. The study was conducted in two phases in order to coincide with the historical peak of the malaria transmission season one-two months after the main rainy season: schools in the southern zones of Borena, Guji and Bale were surveyed in May 2009, while schools in all other zones of Oromia were surveyed between October and December 2009 (Figure 1).

### Sample size and school selection

Oromia was divided into ecological strata defined according to epidemiologically significant differences in elevation and rainfall, based on classifications used by the Federal Ministry of Health and the World Health Organization (WHO) Ethiopia office (Table 1). Malaria transmission is assumed not to occur in arid areas (<500 mm annual rainfall) and highlands (>2,500 metres) [2] and so these strata were not sampled.

**Table 1:** Sampling stratification used to select schools in Oromia Regional State, Ethiopia, based on ecological zones defined according to epidemiologically significant differences in elevation and rainfall, based on classifications used by the Ministry of Health and WHO Ethiopia office [39]

Stratum description	Elevation **(m asl)**^**1**^	Stratum	Total schools in stratum	Proportion of sample	Schools sampled
Highland, occasional epidemic	2,000-2,500	1	1651	0.1	20
Highland fringe, low unstable transmission	1,750-2,000	2	1209	0.409	73
Highland fringe, high unstable transmission	1,500-1,750	3	1163	0.393	71
Lowland with seasonal transmission (annual rainfall 500-1000 mm)	<1,500	4	389	0.132	24
Lowland with intense transmission (annual rainfall >1000 mm)	<1,500	5	196	0.066	12
Highland	>2,500	6	592	Not included in survey	
Arid lowland (annual rainfall <500 mm)	<1,500	7	4	Not included in survey	

^1 ^Metres above sea level.

A two-stage sampling design was employed, whereby schools (primary sampling unit) were selected using probability proportional to size, then within schools a fixed number of children (secondary sampling units) were randomly selected. Therefore, the number of schools sampled from each ecological zone was proportional to the number of schools in each zone, with the exception of the 'highland occasional epidemic' zone (2,000 - 2,500 metres), which was under-sampled as a result of low expected prevalence and a need to maximize the power of the survey in more stable transmission areas. These criteria excluded one administrative zone from the sampling frame (North Shoa, positioned at >2,500 metres), while the remaining 16 administrative zones in Oromia were included in the survey.

**Figure 2:**
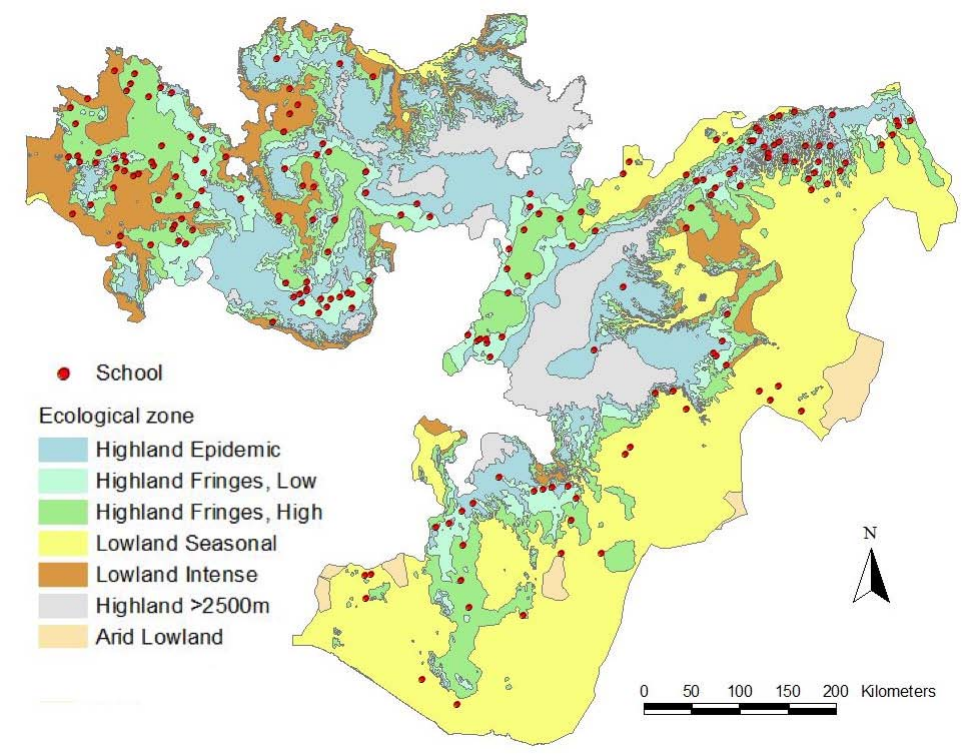
**Distribution of selected schools in relation to ecological zones in Oromia Regional State, Ethiopia**.

The sample size was determined using 95% confidence limits, 80% power and assuming design effect of 2. It was consequently estimated that the sample size for each ecological zone should be 3,925 children from 40 schools to detect a prevalence of 1% with 0.5% precision. Sampling 40 schools from each of five ecological zones would give a final sample size of 20,000 children from 200 schools. It was decided to select schools from ecological zones using probability proportional to population size, due to the uneven distribution of primary schools in Oromia between ecological zones (Table 1 and Figure 2). During the survey, two schools were found to be inaccessible, and one school director refused consent for the survey. No replacement schools could readily be found within time and, thus, 197 schools were included in the final sample.

### Participants

Community sensitization was conducted using a cascade approach. Oromia Regional Health and Education Bureaus gave approval for the study, and official letters were sent to the Bureaus' zonal offices, then to woreda offices and to individual school directors. Information about study procedures and schedule were provided and school directors advised to hold a meeting with the school committee and parents in advance of the designated survey day. Parents who did not want their children to participate in the study were free to refuse participation. Children who were unwilling to participate were excluded from random selection, with written (or thumbprint) assent obtained from selected children before samples were collected. In each school, 10 boys and 10 girls (plus one reserve boy and one reserve girl) aged between five and 18 years were selected from each of grades 2-6 using computer-generated random number tables. If fewer than 110 children were enrolled in the school or present on the survey day, all children aged five to 18 present in the school were included in the survey; this, in some instances, resulted in a small school sample size. Detailed logistical and ethical considerations for this style of school survey have been presented elsewhere [7].

### School survey procedures

Finger-prick blood samples were used to prepare thick and thin blood films for microscopy, and haemoglobin concentration was estimated to an accuracy of 1 g/L using a portable haemoglobinometer (Hemocue Ltd, Angelhölm, Sweden). In addition, blood spots were collected on filter paper for serological analysis at a later date [16,17]. Children were asked a simple set of standardized, pre-tested questions on recent fever, mosquito net use, whether IRS had been conducted in their households, key household socio-economic variables, household construction and education of the child's guardian. Children reporting fever or found to be anaemic (Hb <80 g/L) were tested with a multi-species malaria rapid diagnostic test (RDT) (CareStart^® ^Pf-HRP2 Pan-pLDH, Access Bio, USA) to allow immediate diagnosis and treatment. This test was shown to have 85.6% sensitivity and 92.4% specificity for *Plasmodium falciparum*, and 85.0% sensitivity and 97.2% specificity for *Plasmodium vivax *in Ethiopia [18]. The location of each school was measured in decimal degrees using a hand-held global positioning device (eTREX, Garmin International, Kansas, USA).

### Microscopy quality control

Blood films were fixed and stained at a local health centre after the survey following standard operating procedures [19], and examined after completion of field work by experienced laboratory technicians in Addis Ababa. *Plasmodium *species was recorded, but quantification of parasite density was not conducted. A second reading was carried out for a proportion of blood films, by highly experienced microscopists at the malaria reference laboratory for Oromia Regional State in Adama. Criteria for a second microscopy reading were: slides positive for *Plasmodium *spp. at first microscopy reading; individuals with discrepant microscopy and RDT results; severely anaemic individuals (<80 g/L); and a randomly selected 5% of negative slides. Slides with discrepant results between first and second readings were settled by a third, expert microscopist from the Ethiopian Health and Nutrition Research Institute, the national reference laboratory in Addis Ababa (Additional file 1).

### Satellite-derived environmental data

Elevation was extracted from the shuttle radar topography mission (SRTM) digital elevation model at 1 km^2 ^resolution. Population density was extracted from gridded population of the world (GRUMP) at 5 km^2 ^resolution [20,21]. Land cover type was extracted from the qualitative global land cover map (defined within the UN Land Cover Classification System) using environmental satellite (ENVISAT) mission's Medium Resolution Imaging Spectrometer (MERIS) sensor at 5 km^2 ^resolution. The distance to permanent water bodies was extracted from the World Wildlife Fund (WWF) Global 200 Ecoregions database at 5 km^2 ^resolution [22]. Estimates of enhanced vegetation index (EVI; a proxy for vegetation coverage) and land surface temperature (LST) at 5 km^2 ^resolution were extracted from data provided by the Moderate Resolution Imaging Spectroradiometer (MODIS) instrument aboard the Terra (EOS AM) and Aqua (EOS PM) satellites [23], for the years 2001-2008. Data were processed by a temporal Fourier algorithm to achieve temporal ordination of the data time series whilst preserving important aspects of seasonal variation [24,25]. Environmental variables were linked by location to school-level parasitological data using ArcGIS 9.3 (Environmental Systems Research Institute Inc., Redlands, CA, USA).

### Data analysis

Microscopy results were entered into a Microsoft Excel 2007 spreadsheet (Microsoft Corporation, Seattle, USA). Questionnaire data from school surveys, including RDT results and haemoglobin measurements, were entered into a customized Microsoft Access 2007 database that had been developed to automatically conduct range and consistency checks. Any errors or inconsistencies were corrected with reference to the original paper forms. Survey data were exported from Access and Excel into a combined dataset in STATA 9.0 (Stata Corporation, College Station, TX, USA) for cleaning. Point prevalence maps were developed in ArcGIS 9.3.

Individuals aged over 18 years (n = 260) or with missing parasitological data (n = 270) were excluded from the school survey analysis. Anaemia was defined according to WHO classifications, adjusted by age and elevation [26]. The number, gender ratio and age distribution of children included was described, with breakdown by ecological stratum and survey period. Child age in years was classified into groups: five to nine years, ten to 14 years, and 15 to 18 years. Main outcomes, i.e. any *Plasmodium *infection, *P. falciparum *and *P. vivax *infection individually and anaemia, were presented with binomial 95% confidence intervals (CI) by sex, age group, ecological stratum and survey period. Use of malaria prevention measures, specifically LLINs and IRS, were presented by sex, age group, ecological stratum and survey period with binomial 95% CI, with associations tested using Chi squared test.

Crude univariate associations between outcomes (i.e. *P. falciparum *or *P. vivax *infection) and individual covariates were assessed by random effects logistic regression to control for clustering of infection by school. Full multivariable models to describe association between LLIN use or IRS with *Plasmodium *infection were developed, using zero inflated Poisson (ZIP) models to account for the large proportion of schools with zero prevalence. ZIP models were favoured over standard Poisson models on the basis of the Vuong test [27].

Tests for associations between school-level prevalence and environmental covariates were performed using grouped logistic regression models taking into account clustering within schools. All significant covariates (p < 0.1) were subsequently included in full multivariable models, and non-significant (p < 0.5) covariates excluded sequentially in order of least significance to generate minimal adequate models. Excluded covariates were retested in the minimal model to confirm lack of significance. Bayesian spatial multivariate models were then developed in WinBUGs version 1.4 (MRC Biostatistics Unit, Cambridge and Imperial College London, UK) to explicitly model unexplained spatial correlation between schools. The number of examined and slide-positive individuals for each species at each survey location were modelled as binomial outcomes, including covariates as described above and a geostatistical random effect that modelled spatial correlation using an isotropic, stationary exponential decay function [28].

To further investigate the distribution of *P. falciparum *and *P. vivax *in Oromia, the existence of spatial clusters of high malaria prevalence were investigated using Kulldorff's spatial scan statistic (version 7.0.2; SaTScan software [29]). A Poisson model was used, under the null hypothesis that the expected number of cases for each area was proportional to its population size. The rate ratio was defined as the observed to expected cases; significance of identified clusters was tested by likelihood ratio, based on 9,999 Monte Carlo simulations.

### Ethical considerations

This study received ethical approval from the national health research ethics review committee of the Ethiopian Science and Technology Ministry (RDHE/2-89/2009). Approval for the study was given by the Oromia Regional Health Bureau and the Oromia Regional Education Bureau.

Written consent for the survey was provided by each school director, but parents maintained the right to withdraw their child from the survey. Each child selected for inclusion was required to provide written assent (or thumbprint) after having the procedures explained. Schools where the director refused consent were not included in the school survey, and pupils refusing assent were excluded. Individuals with a positive malaria RDT were treated according to Ethiopian national guidelines [30]. Individuals with haemoglobin <80 g/L were provided with a two-week dose of ferrous sulphate tablets and instructions on how to take this medication, and advised to attend the health centre for follow-up.

## Results

### School survey participants

A total of 21,166 children, age five to 18 years (median 11, inter-quartile range 9-12 years), from 197 rural primary schools in Oromia took part in the survey, with a similar number of boys and girls included (53.2% male). A mean 106 children were enrolled from each school (range 43-112). Blood films from 267 children were missing or unreadable. Consequently, these individuals were excluded from analysis, leaving 20,899 children (98.7%).

### Reported use of malaria interventions

Overall, 46.0% (95% confidence interval (CI): 45.3-46.7%) of school children reported using a LLIN the previous night. In locations where the school elevation exceeded 2,000 m, 42.0% (95% CI: 39.7-43.6) of children reported using a LLIN; however, this may be in part attributable to kebeles with large altitude ranges still falling within the National Malaria Control Programme (NMCP) objectives to target areas under 2,000 m with LLIN distribution [10]. Reported LLIN use was lower amongst males than females (43.4% vs. 49.0%, p < 0.001) and amongst children aged 15-18 years than other age groups (39.4% vs. 46.4% for five to nine years and 46.6% for 10-14 years, p < 0.001).

### Malaria and anaemia

The overall prevalence of *Plasmodium *infection was 0.56% (95% CI: 0.46-0.67%). Of children with *Plasmodium *infection, 52.1% were infected with *P. falciparum*, 47.0% with *P. vivax *and 0.9% with mixed infections. Mixed infections were not analysed separately but included with each of the single species infections. The overall prevalence of *P. falciparum *was 0.30% (95% CI: 0.23-0.38%) and *P. vivax *0.27% (95% CI: 0.20-0.35%). Only 18% of children with *P. falciparum *reported fever on the day of the survey; however, 72% had had fever in the past month. A greater proportion of *P. vivax *infections were sub-clinical, with only 7% of children reporting fever on the survey day, but 56% reporting to have felt fever in the past month. In total, 17.6% of children were anaemic, and the mean haemoglobin concentration was found to be 132.8 g/L (95% CI: 132.6-133.0).

**Figure 3:**
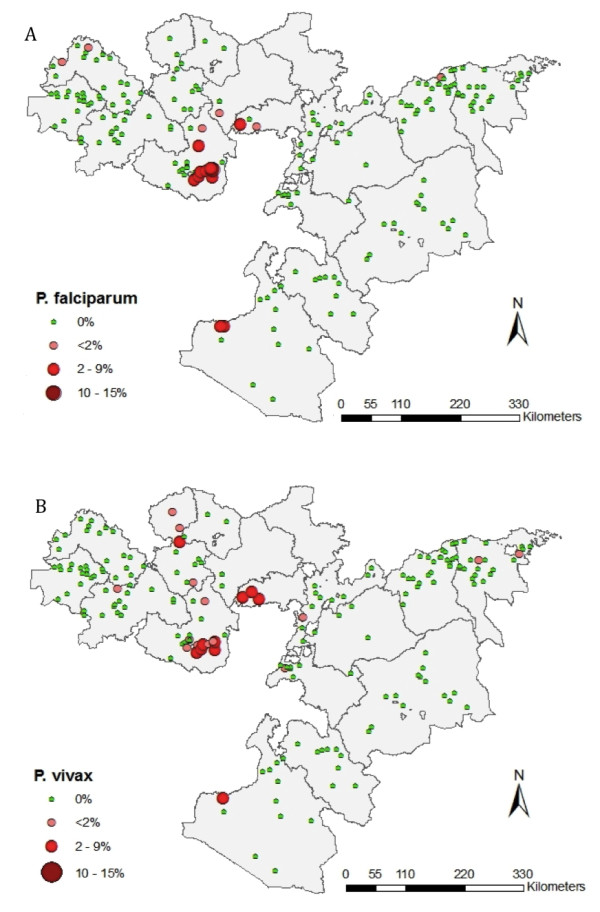
**Prevalence of (a) *Plasmodium falciparum *and (b) *P. vivax *infection by school, Oromia Regional State, Ethiopia, 2009**.

Figure 3 shows the geographical distribution of *P. falciparum *and *P. vivax *prevalence by school. Thirty schools (15%) were found to have at least one child with *Plasmodium *spp. infection on the day of the survey (17 *P. falciparum *and 24 *P. vivax*), and prevalence by school ranged between 0 and 14.5%. All schools with detectable infection were located at an elevation between 1,183 and 2,187 metres above sea level. The median time taken to walk to school, an indicator of distance the child lives from school, was 30 minutes (range 0-240 minutes). The schools with highest prevalence of infection were found in Jimma and South West Shoa administrative zones (Figure 3).

**Figure 4:**
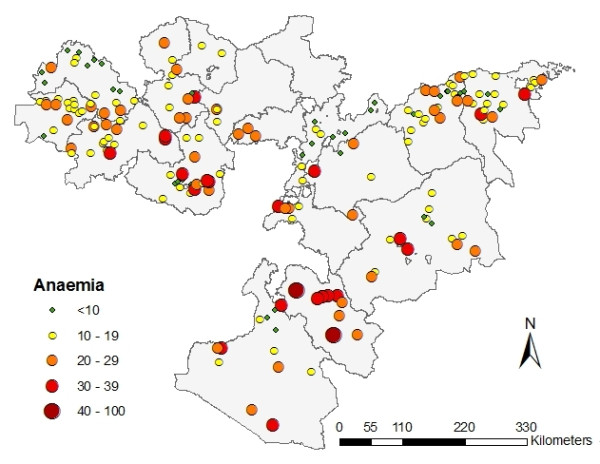
**Prevalence of anaemia by school, Oromia Regional State, Ethiopia, 2009**.

Malaria infections were found in all five ecological strata, with highest prevalence found in the stratum defined as highland fringes with low transmission (Table 2). The proportion of infections due to *P. falciparum *was variable between strata: from 10% in highland epidemic to 86% in lowland seasonal (p = 0.001). There was strong evidence that the *P. falciparum *and *P. vivax *rates differed by ecological zone (both p < 0.001). Prevalence of anaemia varied markedly between schools (Figure 4), ranging between 0.9% and 51.4%. Guji administrative zone was seen to have consistently high levels of anaemia at all schools sampled, while the highest prevalence of anaemia was found in the ecological stratum defined as lowland with seasonal malaria (Table 2).

**Table 2:** Prevalence of *P. falciparum *(*P. f.*)*, P. vivax (P. v.) *and anaemia among primary school children in 197 schools in Oromia Regional State, Ethiopia in 2009, by sex, age group, survey phase and malaria transmission zone

	**N**^**1**^	*Plasmodium* prevalence, % (95% CI)	% due to *P. f.*	Species prevalence, % (95% CI)		Anaemia, % (95% CI)
					
				*P. f.*	*P. v.*	
Total	197/20,899	0.6 (0.5-0.7)	0.53	0.3 (0.2-0.4)	0.3 (0.2-0.3)	17.6 (17.0-18.1)
						
Male	11,038	0.6 (0.4-0.7)	0.57	0.3 (0.2-0.4)	0.2 (0.2-0.3)	19.2 (18.4-19.9)
Female^2^	9,731	0.6 (0.4-0.7)	0.48	0.3 (0.2-0.4)	0.3 (0.2-0.4)	15.7 (15.0-16.5)
						
5-9 yrs	5,471	0.4 (0.2-0.6)	0.77	0.4 (0.3-0.6)	0.1 (0.05-0.2)	18.8 (17.8-19.9)
10-14 yrs	13,890	0.6 (0.5-0.7)	0.44	0.3 (0.2-0.4)	0.3 (0.2-0.4)	16.4 (15.7-17.0)
15-18 yrs^3^	1,400	0.4 (0.2-0.9)	0.50	0.2 (0.04-0.6)	0.2 (0.04-0.6)	23.4 (22.2-26.7)
						
Phase 1	36/3,779	0.4 (0.2-0.6)	0.86	0.3 (0.2-0.6)	0.05 (0.00-0.2)	22.4 (21.1-23.8)
Phase 2	161/17,120	0.6 (0.5-0.7)	0.48	0.3 (0.2-0.4)	0.3 (0.2-0.4)	16.5 (15.9-17.1)
						
Highland epidemic	22/2,358	0.4 (0.2-0.8)	0.10	0.04 (0.00-0.2)	0.4 (0.2-0.7)	14.7 (13.3-16.1)
Highland fringes, low transmission	69/7,246	1.1 (0.8-1.3)	0.55	0.6 (0.4-0.8)	0.5 (0.3-0.7)	16.3 (15.5-17.2)
Highland fringes, high transmission	66/7,018	0.1 (0.06-0.2)	0.56	0.07 (0.02-0.2)	0.06 (0.01-0.1)	17.2 (16.4-18.1)
Lowland seasonal	24/2,540	0.5 (0.3-0.9)	0.86	0.5 (0.2-0.8)	0.08 (0.01-0.3)	24.0 (22.4-25.7)
Lowland intense	16/1,737	0.4 (0.2-0.8)	0.14	0.06 (0.00-0.3)	0.3 (0.1-0.8)	18.7 (16.9-20.6)

^1 ^Number of schools surveyed/children tested

^2 ^Sex data missing from 130 records

^3 ^Age data missing from 138 records

### Risk factors for malaria and anaemia

There were no statistical differences in *Plasmodium *infection by sex or by age (Additional file 2), whereas anaemia was more common among males than females (19.2% vs. 15.7%, p < 0.001), and in children aged 15-18 years (p < 0.001). From crude univariate analysis (Additional file 2), anaemia was found to be strongly associated with *P. falciparum *infection. History of fever in the previous month was associated with both *P. falciparum *and *P. vivax *infection, as well as with anaemia. IRS and LLIN use were found to be associated with increased odds of infection. Odds of *P. falciparum *and *P. vivax *infection were associated with forested areas rather than cultivated land. Some associations with vegetation (EVI) and temperature (LST) were seen, but the effect was small. In multivariate ZIP models, LLIN use was no longer found to be associated with either *P. falciparum *or *P. vivax *infection. The association between IRS in the household and increased risk of infection, however, remained in multivariate models: IRR of 2.91 for *P. falciparum *(95% CI: 1.36-6.21, p = 0.006) and IRR of 3.92 for *P. vivax *(95% CI: 1.82-8.45, p < 0.001).

### Spatial analysis

Bayesian multi-variable modelling suggested there were no significant associations between *P. falciparum *or *P. vivax *prevalence by school and population density, elevation or land cover class, once residual spatial correlation was accounted for. Parameters associated with the magnitude and timing of seasonal changes in LST and EVI did show some association with *P. falciparum *and *P. vivax *infection; although these covariates did not improve model fit (Table 3). The variance of the school-level random effect (σ^2^_school_; which indicates a propensity for clustering) was large for both species. Similarly, for both species the distance at which spatial correlation dropped to below 5% was very large (in excess of 200 km) when compared with other spatial models of malaria infection [21]. This suggests a slow decline of spatial correlation with distance at larger scales, and is likely a consequence of large areas of zero prevalence across Oromia Regional State.

**Table 3:** Fitted parameters in Bayesian multivariate models for *P. falciparum *and *P. vivax *among school children in Oromia Regional State, Ethiopia in 2009, with and without spatial components

	Non-spatial model		Spatial model	
		
	Parameter	95% BCI	Parameter	95% BCI
***P. falciparum ***				
**Without covariates**				
σ^2^_school_	13.09	5.78-25.4	10.0	2.27-25.0
Range of spatial correlation (km)	-	-	271	132-794
DIC (model fit)	120.1	-	113.0	-
**With covariates**				
LST: bi-annual phase	OR: 2.11	1.17-4.19	OR: 2.03	0.96-4.00
σ^2^_school_	11.21	5.32-22.4	8.43	2.89-23.9
Range of spatial correlation (km)	-	-	244	119-758
DIC (model fit)	121.4	-	113.4	-
***P. vivax ***				
**Without covariates**				
σ^2^_school_	6.31	2.96-13.17	7.03	2.30-19.95
Range of spatial correlation (km)	-	-	321	154-927
DIC (model fit)	162.3	-	145.5	-
**With covariates**				
EVI: bi-annual amplitude	OR: 1.93	0.91-4.25	OR: 2.06	0.74-4.92
LST: tri-annual phase	OR: 2.02	0.86-4.26	OR: 1.34	0.44-3.36
σ^2^_school_	6.09	2.98-11.7	8.10	2.32-29.9
Range of spatial correlation (km)	-	-	355	162-1,589
DIC (model fit)	159.8	-	146.3	-

OR, odds ratio; 95% BCI, Bayesian credible interval; σ^2^_school _is the variance of RE; the range of spatial correlation is the distance at which correlation between sites <5%; DIC is deviance information criterion, a measure of model fit where lower values indicate better fit

**Figure 5:**
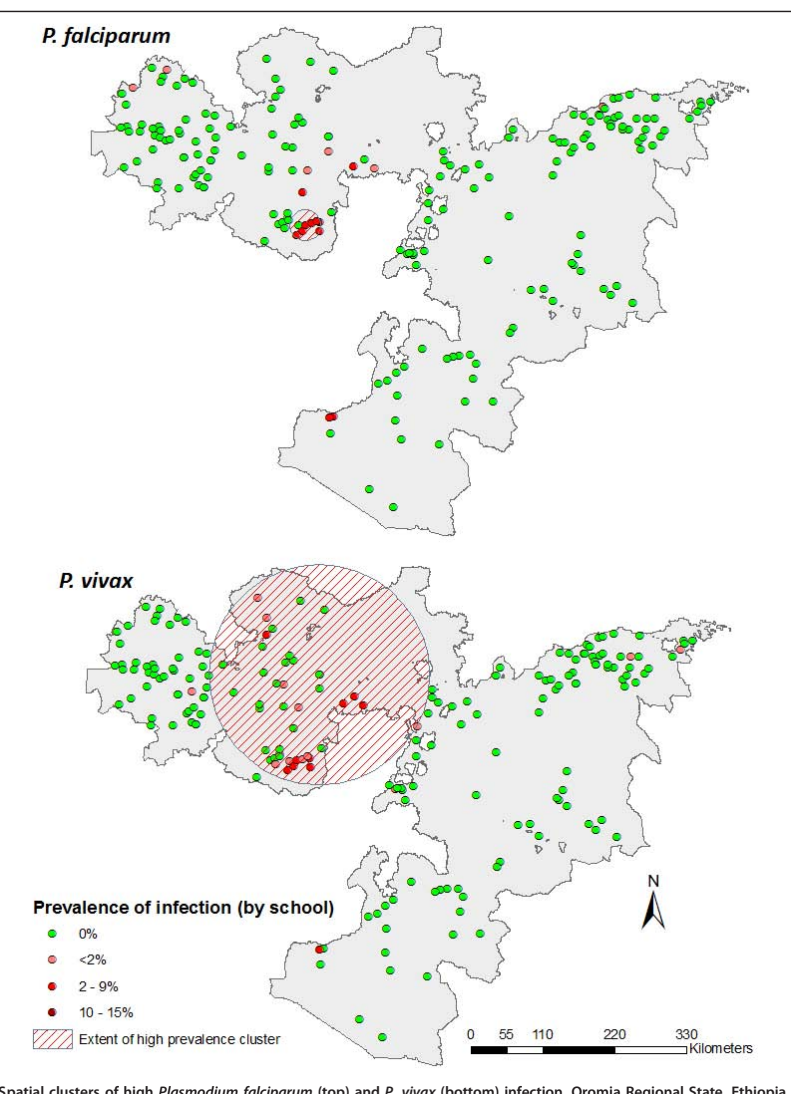
**Spatial clusters of high *Plasmodium falciparum *(top) and *P. vivax *(bottom) infection, Oromia Regional State, Ethiopia, 2009**.

SaTScan analysis identified two clusters of high prevalence of *P. falciparum *and *P. vivax *infection (Figure 5). A small cluster was found for *P. falciparum *infection, with a radius of 23.3 km. The cluster contained 872 children from eight schools, with 40 *P. falciparum *infections found compared to an expected number of 2.6; the relative risk of infection in the cluster being 41.9 times higher than outside of the cluster (p = 0.001). A significant cluster was seen for *P. vivax *infection, which with a radius of 169.0 km, was larger than the *P. falciparum *infection cluster. This cluster included 4,782 children from 44 schools, with 49 *P. vivax *infections found compared to an expected number of 12.5. The relative risk of infection in this cluster was 55.1 times higher than outside of the cluster (p = 0.001). Parameters for seasonal changes in EVI and LST showed some association with presence inside the *P. falciparum *and *P. vivax *infection clusters, while proximity to water was associated with location inside the *P. vivax *cluster (OR = 0.13, p = 0.001).

## Discussion

This first application of school-based surveys to inform targeting of malaria interventions in Ethiopia has revealed a comparable prevalence of *Plasmodium *to that found in the 2007 national MIS survey [12], but lower than that reported in a 2006 survey conducted by the Carter Center (prevalence 4.1%) [11]. The study presented here found *P. vivax *prevalence to be comparable to that of *P. falciparum*, and highlighted the marked spatial heterogeneity in infection observed in Oromia and, indeed, Ethiopia. Furthermore, the results are consistent with other cross-sectional study findings [11], with malaria cases found above 2,000 m, i.e. the current NMCP boundary for classification of an area as malarious and determining inclusion in control activities such as IRS and LLIN distribution [10].

Risk factors for *P. falciparum *infection identified by crude univariate analysis include history of fever and anaemia. Only fever history was, however, associated with *P. vivax *infection. These findings indicate that fever in the previous month is predictive of malaria due to both species, but there remained a high proportion of identified infections that were asymptomatic. Cross-sectional surveys from a range of transmission settings have shown that a strong association exists between *P. falciparum *and reported fever [31]. In low transmission settings, it is often assumed that lack of acquired immunity in the population will cause all *Plasmodium *infections to elicit clinical symptoms, but the current findings dispute this. Since parasite density was not calculated in this study, it is not possible to determine if asymptomatic infections were due to very low parasite density. Asymptomatic infections will contribute to ongoing transmission in a community, but are unlikely to be detected or treated in a context where only individuals feeling unwell access diagnostic services. If Ethiopia is to achieve focal malaria elimination in areas of current low, unstable transmission, alternative strategies must be used to identify and treat asymptomatic infections and halt transmission. In São Tomé and Príncipe, for example, mass screening by means of cross-sectional country-wide surveys, wherein all residents were screened with a RDT and RDT-positive individuals were treated with an ACT, has contributed to recent dramatic reductions in malaria transmission [32].

Absence of age-dependency for infection in the current findings is consistent with lack of acquired immunity among individuals living in low malaria transmission settings [33], and findings from other surveys in Ethiopia [11]. Prevalence of anaemia was found to be higher in the present instance than in the 2005 national school health survey [34], but lower than in the 2005 Demographic Health Survey [4]. Increased odds of anaemia in males, however, was a common finding in the 2005 national school survey, and has been reported from other countries [35]. Males were also less likely to sleep under a LLIN, which may result in more frequent exposure to *Plasmodium *infection and resultant anaemia. Overall, these findings indicate that iron supplementation should be considered as a possible school health strategy, targeting boys and girls.

While documented scale-up in LLIN distribution and coverage in Ethiopia has been very successful [36], the present study shows that use of LLINs remains less than optimal among school-age children. This is consistent with other studies indicating that children of school-age are often the least likely to have access to mosquito nets owned by the household [37], as well as other data from Ethiopia indicating that net use does not directly correspond with net ownership [38]. While possession of LLINs in a household will exhibit some indirect protective effect for individuals not sleeping under the net, Ethiopia's policy of universal coverage with LLINs in malaria risk areas [39] must be fully implemented in order to fully contribute to transmission control. There is also a need for additional behaviour-change activities linked to LLIN distribution campaigns and the routine health extension programme, to ensure consistent use of LLINs [40,41].

Somewhat surprisingly, LLIN use was associated with increased odds of malaria in crude univariate analysis. However, multivariate models did not find such association. Previous cross-sectional studies have found that net use is protective against malaria among school-aged children [42,43], and other surveys found that a protective effect against malaria was linked with the number of nets per household [11]. IRS, as reported by children to have been conducted in their house, was found to be associated with increased risk of both *P. falciparum *and *P. vivax *infection in multivariate models. The lack of protective effect of IRS in these findings may be a result of near-universal resistance to DDT (1,1,1-dichloro-2,2-bis(p-chlorophenyl)ethylene) in Ethiopian anopheline mosquitoes (Reithinger *et al.*, unpublished). The most likely explanation for this association between IRS and increased malaria risk is that the NMCP targets IRS to locations of known malaria endemicity; therefore living in a location where IRS is conducted is predictive of being in a malarious area.

The current surveys found a greater proportion of *Plasmodium *infections due to *P. vivax *than previously described in Ethiopia [11,12], with *P. falciparum *and *P. vivax *in equal proportion overall but *P. vivax *dominating in the highland epidemic ecological stratum (90%). The variation in species distribution may be a result of increased use of artemisinin-based combination therapy and *P. falciparum-*detecting RDTs at peripheral health facilities, impacting on transmission of *P. falciparum *and changing the epidemiology of this parasite in Ethiopia. Alternatively, the findings may simply be due to the highly variable and unstable transmission setting, where increased *P. vivax *cases may be a result of focal epidemics in highland areas at the time of the survey, or a result of the tendency for *P. vivax *to cause long-term chronic infections and show less seasonality in transmission than *P. falciparum *[9]. The recent adoption of multi-species RDTs at health posts across Ethiopia will greatly improve the diagnosis and treatment of *P. vivax *infections. These infections are known to cause morbidity, including anaemia, malnutrition and respiratory distress [44,45], but are likely to have been under-diagnosed in the past due to use of *P. falciparum*-detecting RDTs. Challenges remaining in control of *P. vivax *include examination of drug-efficacy and potential adjustment of national policy, in light of identified foci of chloroquine resistance [46-49], as well as strategies for diagnosing and clearing asymptomatic *P. vivax *infections. Furthermore, similar to other settings, it is likely that as prevalence of *P. falciparum *in Ethiopia is reduced by effective control interventions, the burden of malaria attributable to *P. vivax *will increase [50,51].

It is the commonly held belief that in low transmission settings, a high proportion of children with malaria would be symptomatic and therefore absent from school. The present findings, however, indicate that although self-reported fever during the previous month is predictive of *Plasmodium *infection, only a minority of parasitaemic individuals identified in schools reported any fever on the day of the survey. Similar high proportions of asymptomatic *Plasmodium *infections have been found in other low transmission settings [52]. Attempts were made to ensure that all eligible children enrolled at each school were included in random sampling, but we expect a proportion of enrolled students were absent on the survey day. Provided that the underestimate of true parasite prevalence estimated from school surveys is consistent, this methodology can still be applied to collect valid epidemiological data from schools. Further investigation of the contribution of malaria to school absenteeism should be conducted to evaluate the population representativeness of parasite rates from school-based surveys.

The poor sensitivity of microscopy to detect low-density *Plasmodium *infections [53] may have affected the outcome of this study. The difficulties in correctly identifying low-density infection may have contributed to the discrepancy in microscopy results between standard and expert examination of blood films. Furthermore, these discrepancies indicate a need to implement a rigorous quality assurance system within the routine laboratory diagnostics system for malaria in Ethiopia, or alternatively, to expand the use of RDTs beyond community-level health care. Molecular techniques, such as polymerase chain reaction (PCR), have a lower detection threshold for *Plasmodium *than microscopy [52,54], and may be a more sensitive diagnostic tool in a population where low-density infections are expected. Unfortunately, PCR remains suitable only in a research context, and not as a routine diagnostic tool for malaria.

The current study was unable to create a valid model, based on environmental covariates, to predict malaria endemicity across Oromia, because strong environmental predictors for location of transmission foci were lacking. Risk mapping using similar strategies had previously been successful in Afghanistan, with a comparable prevalence of infection (0.49%) [55]. This inability to develop a risk map based on environmental correlates only indicates that there are additional factors contributing to transmission that were not captured in modelled data. It may also be a result of the spatial and temporal variability of transmission, not adequately captured by a cross-sectional survey approach and microscopy diagnosis. Although it was not possible to determine the exact altitude at which infection was acquired, data indicate that most children live close to the school (median 30 minutes' walk). Therefore, it was assumed that children's homes, the site where infection is likely to have occurred, is at a similar altitude to the school and based on this assumption it was possible to identify two clusters of infection in Oromia, using a method that has successfully described hotspots of malaria at small spatial scale in Kenya and Sudan [56,57].

Identification of all areas where malaria transmission is ongoing may be possible using an alternative diagnostic method where IgG antibodies to *Plasmodium *are detected using an enzyme-linked immunosorbent assay, reflecting exposure to infection over a longer time period. This method has been used successfully in other low and unstable transmission settings [53,58,59]. Alternatively, routinely reported malaria case data from health facilities has been used to model malaria transmission [60], but these data are subject to bias including incomplete recording and reporting, inconsistent quality of diagnostic services and variable access to health facilities across populations and localities. It may be possible to marry parasitological survey data and routine facility data to capture a reliable estimation of malaria transmission levels, and use these combined data to develop a risk map. This approach requires further investigation to ensure comparability between locations, and representativeness of the underlying population.

While the current cross-sectional surveys have provided data regarding the *Plasmodium *parasite rates among children attending school, there is a need to conduct a rigorous comparison to indicators determined from standard community surveys, such as MISs. This will determine if findings from school-based surveys are representative of all school-aged children in a community or, indeed, the whole community; if representative, school-based surveys could become an alternative survey method to the more costly and labour-intensive community surveys. While it is not expected that there are differences in risk of infection by age in Ethiopia, there is a need to further explore what proportion of school-absenteeism is due to malaria, as well as whether there are differences in malaria risk between enrolled and non-enrolled children. These findings will define the potential role of schools in malaria surveillance, monitoring and control in Ethiopia and other low transmission settings. Envisaged roles of schools in malaria surveillance could be to provide data on coverage of major interventions and parasite prevalence during routine school surveys, and to alert service providers of epidemics using information on school-absenteeism and from active case finding [6].

## Conclusions

Results of cross-sectional school surveys in Oromia demonstrated marked spatial heterogeneity in malaria. Although several foci of infection were identified, large areas appear to be non-endemic for malaria. While these findings allow malaria control interventions to be targeted to identified endemic areas, this likely does not reflect the true extent of malaria in Oromia. Research is ongoing to further validate the use of school surveys in identifying transmission foci, as well as to investigate other potential uses of schools in malaria surveillance including monitoring and evaluation of control programme implementation.

## Competing interests

The authors declare that they have no competing interests.

## Authors' contributions

RA coordinated project management, data collection, analysis and developed the draft manuscript. TK, GT and DY were responsible for fieldwork supervision and project coordination and contributed to the final manuscript. RP contributed to data analysis and undertook spatial modelling. SB, RR and JK were responsible for the study design, interpretation and scientific guidance. All authors read and approved the final manuscript. The opinions expressed in this paper are those of the authors and may not reflect the position of their employing organisations nor of their funding sources.

## Supplementary Material

Additional file 1 **Microscopy results quality control flowchart**. Click here for file

Additional file 2 **Univariate analysis for associations between *Plasmodium falciparum *and *P. vivax *and potential risk factors among school children in Oromia Regional State, Ethiopia in 2009, adjusting for clustering within schools**. Click here for file
