# Supplementary figures and images for: School-based surveys of malaria in Oromia Regional State, Ethiopia: a rapid survey method for malaria in low transmission settings
Source: Malar J. 2011 Feb 3;10:25. doi: 10.1186/1475-2875-10-25 (PMC3039636; doi:10.1186/1475-2875-10-25)

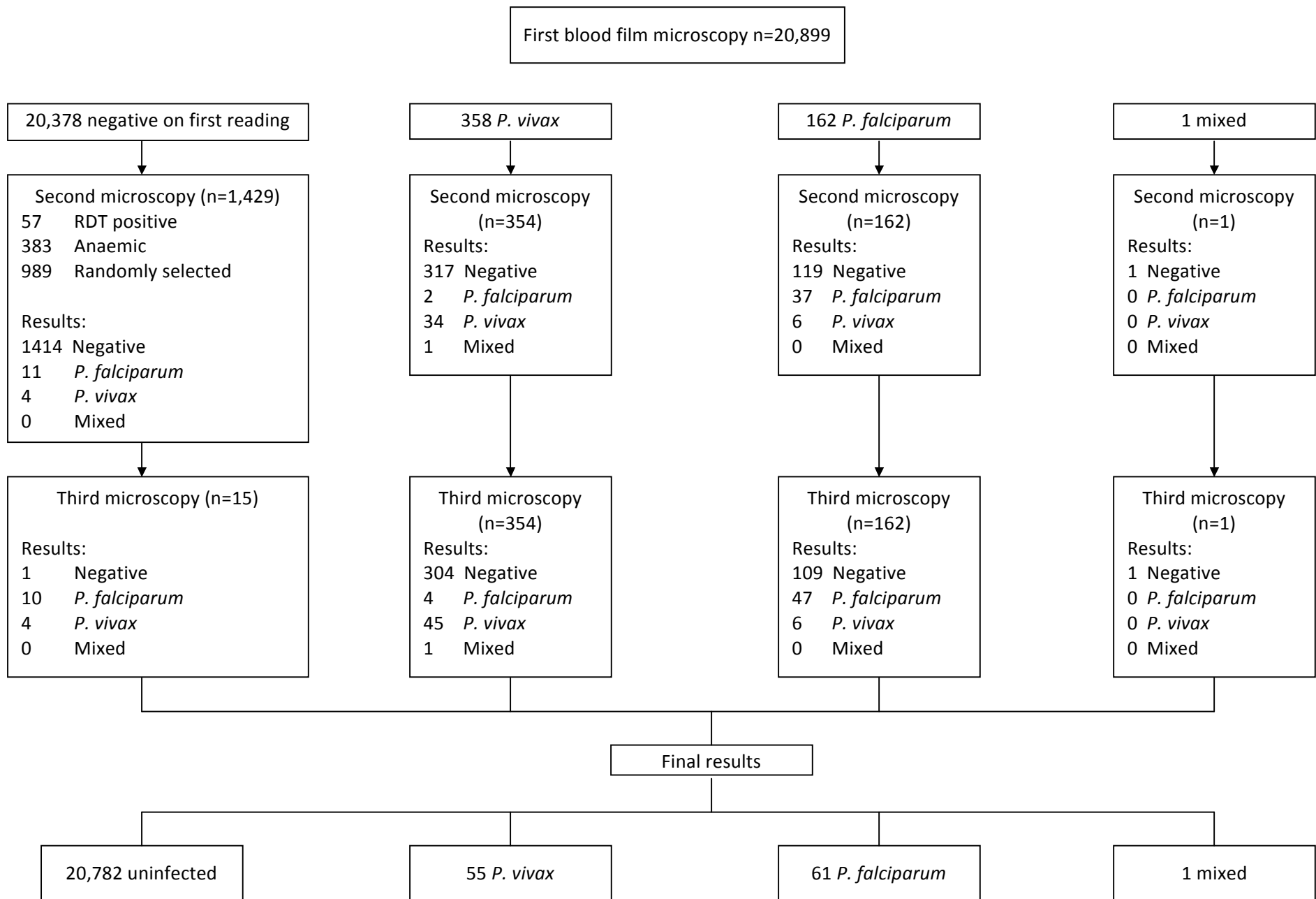

Supplement: Additional file 1 — Microscopy results quality control flowchart. [file 1475-2875-10-25-S1.PDF]
