# Supplementary material for: School-based surveys of malaria in Oromia Regional State, Ethiopia: a rapid survey method for malaria in low transmission settings
Source: Malar J. 2011 Feb 3;10:25. doi: 10.1186/1475-2875-10-25 (PMC3039636; doi:10.1186/1475-2875-10-25)
Supplement: Additional file 2 — Univariate analysis for associations between Plasmodium falciparum and P. vivax and potential risk factors among school children in Oromia Regional State, Ethiopia in 2009, adjusting for clustering within schools. [file 1475-2875-10-25-S2.DOC]

**Univariate analysis for associations between *Plasmodium falciparum* and *P. vivax* and potential risk factors among school children in Oromia Regional State, Ethiopia in 2009, adjusting for clustering within schools**

|  | *P. falciparum* | | | *P. vivax* | | |
| --- | --- | --- | --- | --- | --- | --- |
|  | OR^1^ | 95% CI | Wald p | OR | 95% CI | Wald p |
| Sex (female) | 0.88 | 0.57-1.35 | 0.5 | 1.27 | 0.75-2.14 | 0.4 |
| Age (linear, increasing) | 0.89 | 0.74-1.07 | 0.2 | 1.05 | 0.96-1.16 | 0.3 |
| Socio-economic status: |  |  |  |  |  |  |
| Poorest | 1 | - | - | 1 | - | - |
| 2^nd^ | 0.92 | 0.49-1.73 | 0.8 | 1.45 | 0.67-3.11 | 0.3 |
| 3^rd^ | 0.60 | 0.19-1.90 | 0.4 | 0.93 | 0.36-2.38 | 0.8 |
| 4^th^ | 1.14 | 0.49-2.66 | 0.8 | 1.10 | 0.58-2.06 | 0.8 |
| Least poor | 0.69 | 0.23-2.04 | 0.5 | 0.74 | 0.30-1.78 | 0.5 |
| Anaemia | 6.89 | 4.02-11.82 | <0.001 | 1.36 | 0.73-2.54 | 0.3 |
| Fever | 7.89 | 4.26-14.62 | <0.001 | 4.97 | 2.71-9.14 | <0.001 |
| LLIN use | 2.01 | 1.11-3.64 | 0.02 | 1.21 | 0.67-2.18 | 0.5 |
| IRS in home | 2.69 | 1.34-5.42 | 0.005 | 2.83 | 1.28-6.23 | 0.01 |
| Ecozone: |  |  |  |  |  |  |
| Highland epidemic | 1.00 | - | - | 1.00 | - | - |
| Highland fringe, low transmission | 12.8 | 1.22-134.1 | 0.03 | 1.57 | 0.49-5.01 | 0.4 |
| Highland fringe, high transmission | 1.64 | 0.13-20.71 | 0.7 | 0.20 | 0.04-0.96 | 0.05 |
| Lowland seasonal | 8.59 | 0.75-99.05 | 0.09 | 0.24 | 0.30-1.92 | 0.18 |
| Lowland intense | 1.39 | 0.05-38.10 | 0.8 | 0.88 | 0.16-4.87 | 0.8 |
| Enhanced vegetation index (EVI)^2^ |  |  |  |  |  |  |
| Annual amplitude | 1.00 | 0.996-1.01 | 0.8 | 1.00 | 0.99-1.00 | 0.5 |
| Bi-annual amplitude | 1.00 | 0.99-1.01 | 0.6 | 1.01 | 1.00-1.02 | 0.01 |
| Tri-annual amplitude | 0.99 | 0.98-1.01 | 0.5 | 0.99 | 0.99-1.01 | 0.9 |
| Bi-annual phase | 0.99 | 0.99-0.99 | 0.05 | 1.01 | 0.99-1.01 | 0.2 |
| Quarterly phase | 0.99 | 0.99-1.00 | 0.8 | 0.99 | 0.99-0.99 | 0.007 |
| Land surface temperature (LST) |  |  |  |  |  |  |
| Annual amplitude | 1.00 | 0.99-1.00 | 0.3 | 1.00 | 0.99-1.00 | 0.7 |
| Bi-annual amplitude | 0.99 | 0.99-1.00 | 0.9 | 1.01 | 1.00-1.01 | 0.009 |
| Tri-annual amplitude | 0.99 | 0.98-1.01 | 0.23 | 0.99 | 0.98-1.00 | 0.1 |
| Bi-annual phase | 1.00 | 1.00-1.00 | 0.003 | 1.00 | 0.99-1.00 | 0.7 |
| Quarterly phase | 0.99 | 0.99-1.01 | 0.9 | 1.01 | 1.00-1.01 | 0.002 |
| Distance to water (increasing) | 0.67 | 0.41-1.10 | 0.1 | 0.58 | 0.39-0.86 | 0.007 |
| Population density (increasing) | 0.99 | 0.99-1.00 | 0.9 | 1.00 | 0.99-1.00 | 0.2 |
| Land-cover type: |  |  |  |  |  |  |
| Cultivated land | 1 | - | - | 1 | - | - |
| Forest | 3.81 | 1.03-14.08 | 0.05 | 3.58 | 1.26-10.24 | 0.02 |
| Shrubland | 2.65 | 0.32-21.92 | 0.4 | 1.39 | 0.19-10.32 | 0.8 |
| Bare / sparse | *predicts perfectly* | | - | *predicts perfectly* | | - |

^1^ OR = odds ratio; 95% CI = 95% confidence intervals

^2^ Variables for EVI and LST describe the phase and amplitude of annual, bi-annual, tri-annual and quarterly cycles after processing data by a Fourier algorithm.
